# Overexpression of c-*erb*B2 is a negative prognostic factor in anaplastic astrocytomas

**DOI:** 10.1186/1746-1596-5-18

**Published:** 2010-03-23

**Authors:** Sasha Gulati, Borgny Ytterhus, Unn S Granli, Michel Gulati, Stian Lydersen, Sverre H Torp

**Affiliations:** 1 Department of Neurosurgery, St Olavs University Hospital, Trondheim, Norway; 2 Department of Laboratory Medicine, Children's and Women's Health, Norwegian University of Science and Technology, Trondheim, Norway; 3 Department of Pathology and Medical Genetics, St Olavs University Hospital, Trondheim, Norway; 4 Department of Circulation and Medical Imaging, Norwegian University of Science and Technology, Trondheim, Norway; 5 Department of Cancer Research and Molecular Medicine, Norwegian University of Science and Technology, Trondheim, Norway

## Abstract

The epidermal growth factor receptor (EGFR) family, consisting of four tyrosine kinase receptors, c-*erb*B1-4, seems to be influential in gliomagenesis. The aim of this study was to investigate EGFR gene amplification and expression of c-*erb*B1-4 receptor proteins in human anaplastic astrocytomas. Formalin-fixed and paraffin-embedded sections from 31 cases were investigated by standard immunohistochemical procedures for expression of c-*erb*B1-4 receptor proteins using commercial antibodies. EGFR gene amplification was studied by fluorescence in situ hybridization using paraffin-embedded tissues. Two monoclonal antibodies, NCL-EGFR-384 and NCL-EGFR, were used for EGFR detection and they displayed positive immunoreactivity in 97% and 71%, respectively. For c-*erb*B2 detection three monoclonal antibodies, CB11, 3B5, and 5A2, were applied and they displayed positive immunoreactivity in 45%, 100%, and 52%, respectively. Positive immunostaining for c-*erb*B3 and c-*erb*B4 was encountered in 97% and 74%, respectively. The EGFR gene was amplified in 9 out of 31 tumors (29%). After adjusting for age, Karnofsky performance status, and extent of surgical resection, Cox multiple regression analysis with overall survival as the dependent variable revealed that c-*erb*B2 overexpression detected by the monoclonal antibody clone CB11 was a statistically significant poor prognostic factor (P = 0.004). This study shows the convenience and feasibility of immunohistochemistry when determining the expression of receptor proteins in tissue sections of human astrocytomas. The synchronous overexpression of c-*erb*B1-4 proteins in anaplastic astrocytomas supports their role in the pathogenesis of these tumors. Further, c-*erb*B2 overexpression seems to predict aggressive behaviour.

## Background

Anaplastic astrocytomas constitute 4% of all malignant nervous system tumors [1]. Patients with anaplastic astrocytomas face a poor prognosis despite major efforts to improve radiation, chemotherapy, and surgical procedures. Median survival for patients with anaplastic astrocytoma is 3 to 5 years [2]. Age at diagnosis, extent of surgery and Karnofsky performance score (KPS) are established prognostic factors in high grade glioma patients [3].

The astrocytic tumors are prone to progress, and members of the epidermal growth factor receptor (EGFR) family have been linked to this malignant transformation. This receptor family consists of four tyrosine kinase receptors, c-*erb*B1-4, and seems to be influential and involved in tumor cell proliferation, differentiation, cell survival, and angiogenesis [4-9]. Coexpression of c-*erb*B1-4 renders the possibility of dimerization of these receptors, thereby recruiting and enhancing signal transducing pathways [4,7,10]. Due to overexpression of the c-*erb*B1-4 receptor proteins and their location on the surface of neoplastic astrocytes, they are attractive candidates for targeted therapy [11-13]. Current strategies include inhibition of the intrinsic kinase activity by monoclonal antibodies [14-16]. Such treatment, however, requires reliable detection systems for these receptor proteins in tumor tissue. Immunohistochemistry appears as the principal mean to detect these receptor proteins. Although this is a convenient and feasible technique, different staining results can be achieved due to varying sensitivity and specificity of commercial antibodies.

Several studies have to a varying degree shown amplification of the EGFR (c-*erb*B1) gene, located on chromosome 7, in glioblastoma multiforme [17-24]. EGFR gene amplification distinguishes small cell glioblastomas from anaplastic oligodendrogliomas, and it has been shown to be an indicator for resistance to radiotherapy [25,26]. EGFR gene amplification can now simply be evaluated by means of fluorescence in situ hybdridization (FISH). However, there is limited knowledge concerning the occurrence of EGFR gene amplification and the expression of *erb*-receptors in anaplastic astrocytomas [27,28].

This study was an extension of our research on *erb *receptor expression in glioblastomas [8,29], and was designed to investigate the extent of EGFR gene amplification and overexpression in anaplastic astrocytomas. Further, we wanted to explore the expression of other members of the EGFR family in anaplastic astrocytomas and investigate their prognostic significance.

## Patients and methods

All 31 supratentorial human anaplastic astrocytomas were operated at the Department of Neurosurgery, St. Olav University Hospital, Trondheim, Norway, and consecutively collected in the time period 1998 to 2006. Craniotomies were performed under general anesthesia, with the patient's head resting in a Mayfield frame system (OMI, Inc., Cincinnati, OH, USA) attached to a reference frame for neuronavigation. The preoperative data was imported into an ultrasound-based navigation system and used for surgical planning and resection guidance [30]. All patients underwent magnetic resonance imaging (MRI) a few days before and within 72 hours after surgery. The extent of tumor resection was determined by the postoperative MRI scans. Surgical resection was defined as gross total resection, partial resection, or biopsy. A chart review was performed to collect demographic and clinical data that included age, sex, and symptoms at presentation, tumor localization, treatment modalities, and postoperative survival. Preoperative Karnofsky performance status score was retrospectively determined from a routine neurological examination from patient admittance, one to three days before surgery.

Expression of c-*erb*B1-4 receptor proteins was determined by immunohistochemistry using various commercial monoclonal antibodies listed in Table 1. Formalin-fixed and paraffin-embedded sections, 4 μm thick, with representative tumor tissue, were incubated with primary antibodies after antigen retrieval by pressure cooking. An automatized histostainer was used for the immunohistochemcial procedures (Dako Autostainer, Glostrup, Denmark). For visualization of immunoreactivity, DAKO EnVision system was used with diaminobenzidin as chromogene. Sections were counterstained with haematoxylin. Positive controls were included in each staining run.

**Table 1:** *erb*B antibodies used

Antibody	Source	Type	Clone	Reactant	Positive control	Dilution	Incubation time and temperature
NCL-EGFR-384	Novocastra, Newcastle-upon-Tyne, UK	Monoclonal	EGFR.25	EGFR (c-*erb*B1 protein) (internal domain)	Human skin	1:100	60 min. at room temperature
NCL-EGFR	Novocastra	Monoclonal	EGFR.113	EGFR (c-*erb*B1 protein) (external domain)	Human skin	1:10	60 min. at room temperature
c-*erb*B-2	Novocastra	Monoclonal	CB11	c-*erb*B2 protein (internal domain)	SKBR-3 breast cancer cell line	1:40	60 min. at room temperature
c-*erb*B-2	Immunotech, Marseille, France	Monoclonal	3B5	c-*erb*B2 protein (internal domain)	SKBR-3 breast cancer cell line	Ready to use	60 min. at room temperature
c-*erb*B-2	Novocastra	Monoclonal	5A2	c-*erb*B2 protein (internal domain)	SKBR-3 breast cancer cell line	1:50	60 min. at room temperature
NCL-c-*erb*B-3	Novocastra	Monoclonal	RTJ1	c-*erb*B-3 protein	Normal kidney	1:10	Over night at 4°C
c-*erb*B-4/HER4	NeoMarkers (Fremont, CA, USA)	Monoclonal	HFR-1	c-*erb*B-4 protein	Breast carcinoma	1:10	Over night at 4°C

The immunoreactivity was assessed by means of intensity and percentage of immunoreactive tumor cells. Intensity was recorded as 0 (no reaction) to 3 (strong reaction). Fraction of immunoreactive tumor cells was recorded as 0 (no positive cells), 1 (<10% positive cells), 2 (10-50% positive cells), or 3 (>50% positive cells). A staining index was calculated as the product of intensity and fraction of positive tumor cells [8].

EGFR gene copy number was investigated on 4 μm thick paraffin sections by double FISH using the LSI EGFR Dual Color Probe- Hyb Set, Vysis no 32-191053 (Abbott Molecular Inc, IL, US). The first probe hybridized with the EGFR gene (chromosomal 7p12 region). Against the centromeric region a second probe was used which revealed the number of copies of chromosome 7. The sections were deparaffinised using xylene, rehydrated, and pretreated using DAKO solution kit (Histology FISH Accessory kit). The probes were added to the sections, coverslipped, sealed with rubber cement, and placed in a DAKO Hybridizer. The sections and probes were codenatured for 5 min at 73°C, followed by annealing at 37°C over night. After hybridization slides were washed in 0.4 × SSC (with detergent) at 73°C for two minutes followed by one minutes in 2 × SSC at room temperature. Then the sections underwent dehydrating in ethanol three times for 3 min. The slides were counterstained and embedded with a 4,6-diamidino-2-phenylindole/antifade solution (DAKO). FISH signals for each locus-specific FISH probe were assessed under a Nikon Eclipse 90i microscope (Nikon, Tokyo, Japan) using Cytovision software (Applied Imaging, Newcastle upon Tyne, England) equipped with a triple-pass filter (DAPI/Green/Orange). The entire area of tumour tissue was evaluated in each case, and all non-overlapping nuclei were assessed for orange (marker) and green (reference) signals by a pathologist (SHT) blinded to any information about the patients. Using the criteria given by Vysis Inc., for HER-2/neu FISH, EGFR signals to chromosome 7 centromere signals of 2 or greater were considered gene amplification [31].

Statistical analyses were made using SPSS version 16.0 (SPSS Inc., Chicago, IL). Survival time was calculated from date of surgery to date of death. Multiple Cox regression analyses were used to study the association between EGFR receptor protein expression (Staining Index, continuous variable) and survival, adjusting for age at diagnosis (continuous variable), Karnofsky performance status scores (continuous variable), and extent of surgical resection (categorical variable; gross total resection versus partial resection and biopsy). The association between results from the FISH investigations (categorical variables; positive versus negative) and survival were studied in the same manner. Two-sided P-values less than 0.05 were regarded as statistically significant.

The study was approved by the Regional Committee for Medical Research Ethics, and study protocols adhered to guidelines by the Helsinki Convention.

## Results

Thirty-one consecutive patients (13 women and 18 men; mean age, 50.2 yr; age range, 28-78 yr) with anaplastic astrocytomas were included in the study. Clinical features of the study group are presented in Table 2. Gross total tumor resection or partial resection was achieved in 10 (32%) and 16 (52%) patients, respectively. In 5 (16%) patients only biopsies were obtained. Four patients had previously undergone surgical resection of diffuse WHO grade II astrocytomas. Chemotherapy and radiotherapy were provided in 7 and 24 cases, respectively.

**Table 2:** Patient characteristics.

**Case no**.	Age (years)/Sex	Prior brain tumor	KPS	Resection grade	Radiotherapy	Postoperative survival (months)
1	57/M	No	80	Partial	1.8 Gy × 30	16.6
2	34/F	No	80	Partial	1.8 Gy × 30	29.4
3	44/M	Grade II astrocytoma	90	Partial	No	41.5
4	28/F	No	90	GTR	1.8 Gy × 30	55.4
5	57/F	No	70	Partial	1.8 Gy × 30	5.6
6	50/M	Grade II astrocytoma	80	Partial	1.8 Gy × 30	23.7
7	67/F	No	80	Partial	1.8 Gy × 30	12.1
8	39/F	No	70	GTR	N.D.	N.D.
9	53/M	No	70	Partial	1.8 Gy × 30	24.6
10	78/F	No	50	Biopsy	3.0 Gy × 13	9.8
11	34/F	No	80	Partial	1.8 Gy × 30	95.8+
12	42/M	No	70	GTR	1.8 Gy × 30	93.6+
13	78/M	No	50	Biopsy	No	1.9
14	40/M	No	70	GTR	1.8 Gy × 30	53.9
15	47/M	Grade II astrocytoma	90	GTR	No	90+
16	49/M	No	50	Biopsy	No	0.1
17	64/M	No	70	GTR	1.8 Gy × 30	76.1+
18	48/F	No	80	GTR	1.8 Gy × 30	8.3
19	57/F	No	80	Partial	1.8 Gy × 30	63.8+
20	72/M	No	60	Biopsy	1.8 Gy × 30	11.1
21	36/M	No	70	Partial	3.0 Gy × 13	11.2
22	56/F	No	70	Partial	2.0 Gy × 30	17.9
23	53/F	No	80	GTR	2.0 Gy × 30	59.8+
24	44/F	No	80	GTR	No	59.8+
25	37/M	No	80	GTR	No	54.6+
26	58/M	No	80	Partial	1.8 Gy × 30	28.6
27	32/F	No	70	Partial	2.0 Gy × 30	16.2
28	38/M	No	80	Partial	2.0 Gy × 30	35.3+
29	71/M	No	50	Biopsy	3.0 Gy × 13	10.2
30	49/M	No	70	Partial	2.0 Gy × 30	30.6+
31	44/M	Grade II astrocytoma	80	Partial	1.8 Gy × 30	39.4+

M = Male, F = Female, KPS = Karnofsky Performance Status, GTR = Gross total resection, N.D. = No data. (+) denotes that the patient is still alive.

Immunohistochemical and FISH data are presented in Table 3. In general, the immunoreactivity was localized to either the cell membrane or the cytoplasm or both. The distribution of tumor cells overexpressing *erb *proteins was both diffuse and dispersed with intensity varying from weak to strong.

**Table 3:** Immunohistochemical and FISH data

**Case no**.	c-*erb*B-1 (clone EGFR.25) (internal domain)	c-*erb*B-1 (clone EGFR.113) (external domain)	c-*erb*B-2 (clone CB11) (internal domain)	c-*erb*B-2 (clone 3B5) (internal domain)	c-*erb*B-2 (clone 5A2) (internal domain)	c-*erb*B-3	c-*erb*B-4	EGFR gene amplification (FISH)
1	9	9	4	6	6	9	4	Yes
2	9	1	0	9	3	4	2	No
3	6	0	4	3	1	9	0	No
4	6	1	1	9	0	0	0	No
5	0	0	4	6	2	6	4	No
6	1	9	1	4	0	4	0	Yes
7	9	9	0	1	0	0	0	No
8	6	2	0	6	4	6	4	Yes
9	9	9	2	6	0	6	6	No
10	9	9	0	9	1	9	4	Yes
11	4	0	2	6	0	4	4	Yes
12	9	4	0	6	2	9	2	Yes
13	9	2	4	6	1	9	2	No
14	4	0	0	1	0	0	0	No
15	2	1	0	9	1	6	0	No
16	6	0	0	4	0	6	1	No
17	9	2	0	9	4	9	2	No
18	4	1	6	9	1	4	4	No
19	4	0	0	1	0	4	0	Yes
20	6	1	1	6	0	6	1	Yes
21	6	4	1	6	0	4	2	No
22	9	6	2	6	6	4	2	No
23	9	3	0	6	0	1	0	No
24	6	0	0	6	3	6	2	No
25	6	2	1	6	3	9	2	No
26	6	4	6	6	2	4	1	Yes
27	6	0	0	1	0	2	1	No
28	6	1	0	4	0	4	1	No
29	9	6	0	4	0	0	1	No
30	3	0	0	4	0	2	2	No
31	6	1	0	9	3	9	6	No

Median Staining Index	6	1	0	6	1	4	2	

No. of positive cases/Total no. of cases	30/31	22/31	14/31	31/31	16/31	27/31	23/31	9/31
Percentage positivity	97%	71%	45%	100%	52%	97%	74%	29%

Two monoclonal antibodies were used for EGFR detection. NCL-EGFR (clone EGFR.113) is reactive against the external EGFR domain and rendered 22 positive tumors (71%). NCL-EGFR-384 is directed towards the internal domain, and 30 tumors (97%) were positive with this antibody.

**Figure 1:**
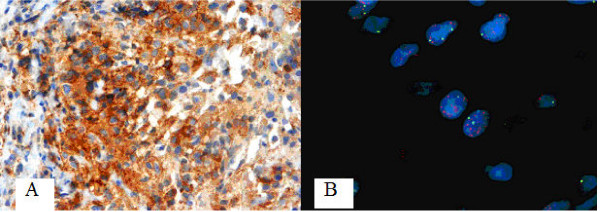
**(A) Positive immunoreactivity for c-*erb*B2 **(clone CB-11) (40×). **(B) FISH analysis showing EGFR gene amplification** (Chromosome 7 centromeric probe in green, EGFR probe in red).

To determine c-*erb*B2 expression, three different monoclonal antibodies were applied. The antibody clones CB11, 3B5, and 5A2 were all reactive against the internal domain and displayed positive immunoreactivity in 45%, 100%, and 52%, respectively (Figure 1A).

C-*erb*B3 immunoreactivity occurred in 27 out of 31 anaplastic astrocytomas (97%), and c-*erb*B4 immunoreactivity was present in 23 out of 31 cases (74%).

The EGFR gene was amplified in 9 out of 31 tumors (29%) assessed by FISH (Figure 1B).

Multiple Cox regression analysis with overall survival as the dependent variable was performed for clinical disease features with expression of c-*erb*B1-4 and EGFR gene amplification (Table 4). After adjusting for age, Karnofsky performance status, and extent of surgical resection, the Cox analysis revealed that c-*erb*B2 overexpression detected by the monoclonal antibody clone CB11 was a statistically significant poor prognostic factor in patients with anaplastic astrocytoma (P = 0.004). Neither overexpression of EGFR, c-*erb*B3, and c-*erb*B4, nor EGFR gene amplification reached significance.

**Table 4:** Results from multiple Cox regression analyses

Variable	Hazard ratio	95% Confidence interval	*P*
c-erbB1 (clone EGFR.113) staining index	1.076	0.92 to 1.26	0.37
Karnofsky performance status	0.913	0.85 to 0.98	0.008
Gross total resection	0.196	0.05 to 0.79	0.022
Age	0.998	0.95 to 1.05	0.95

c-erbB1 (clone EGFR.25) staining index	0.991	0.80 to 1.23	0.93
Karnofsky performance status	0.912	0.85 to 0.98	0.009
Gross total resection	0.199	0.05 to 0.82	0.026
Age	1.007	0.96 to 1.06	0.79

c-erbB2 (clone CB11) staining index	1.642	1.17 to 2.30	0.004
Karnofsky performance status	0.854	0.78 to 0.94	0.001
Gross total resection	0.324	0.08 to 1.38	0.13
Age	0.973	0.92 to 1.03	0.36

c-erbB2 (clone 5A2) staining index	1.036	0.79 to 1.35	0.80
Karnofsky performance status	0.910	0.85 to 0.98	0.009
Gross total resection	0.186	0.05 to 0.78	0.021
Age	1.005	0.96 to 1.06	0.85

c-erbB2 (clone 3B5) staining index	1.018	0.84 to 1.23	0.86
Karnofsky performance status	0.912	0.85 to 0.98	0.009
Gross total resection	0.190	0.05 to 0.77	0.020
Age	1.006	0.96 to 1.06	0.81

c-erbB3 (clone RTJ1) staining index	0.975	0.83 to 1.15	0.76
Karnofsky performance status	0.914	0.85 to 0.98	0.010
Gross total resection	0.204	0.05 to 0.81	0.024
Age	1.008	0.96 to 1.06	0.74

c-erbB4 (clone HFR-1) staining index	0.902	0.68 to 1.20	0.48
Karnofsky performance status	0.907	0.85 to 0.97	0.005
Gross total resection	0.178	0.05 to 0.72	0.015
Age	1.003	0.96 to 1.05	0.90

EGFR gene amplification	0.689	0.23 to 2.11	0.51
Karnofsky performance status	0.919	0.86 to 0.99	0.021
Gross total resection	0.175	0.04 to 0.73	0.017
Age	1.013	0.96 to 1.07	0.63

Multiple Cox regression analyses were used to study the association between c-*erb*B1-4 expression and survival, adjusting for age at diagnosis, Karnofsky performance status scores, and extent of surgical resection. The association between EGFR amplification and survival was studied in the same manner.

## Discussion

In this study we have found abundant co-expression of c-*erb*B1-4 receptor proteins in anaplastic astrocytomas. Cox multiple regression analysis showed that c-*erb*B2 expression detected by the monoclonal antibody clone CB11 was a statistically significant poor prognostic factor. Additionally, the EGFR gene was amplified in 29% of the tumors.

The overexpression of EGFR in anaplastic astrocytomas is in line with our earlier report and other studies [24,28,32-34]. Interestingly, the antibody reactive against the cytoplasmic domain unveiled far more immunoreactive tumors than the antibody against the external domain (97% versus 71%). A similar observation was made in our recent report on glioblastomas [8]. A possible explanation might be that the extracellular part of the EGFR is more vulnerable to fixation and tissue processing. It may also be due to proteolytic cleavage and shedding of EGFR as is described for c-*erb*B2 [35,36]. In any case, further studies must be undertaken to decide which antibody is best suited for standardized EGFR immunohistochemical detection. Such investigations require alternative assays for protein expression, such as protein blotting.

Amplification of the EGFR gene occurs in up to 50% of human glioblastomas, whereas the reported frequency in anaplastic astrocytomas varies considerably between none to about one third of the cases [23,24,28,32,37-40]. Different techniques and definitions of gene dosage may explain these conflicting data. In this regard, FISH analyses have turned out to be a simple and reliable method [28,32]. In our series of anaplastic astrocytomas this approach revealed a relatively high frequency of EGFR gene amplification with 29% of the tumors exhibiting this genetic rearrangement. It seems this genetic event is not solely confined to glioblastomas, and it may be coupled to the malignant progression of anaplastic astrocytomas. It remains to be shown whether this gene amplification is a cause or a consequence of tumor progression. Nevertheless, it may be involved in the oncogenesis of both primary and secondary glioblastomas. Overexpression of EGFR in astrocytomas can also occur without gene amplification [8,28,32,37,39], suggesting other mechanisms at transcriptional and translational levels. An amplified EGFR gene may lead to expression of mutant EGFR (EGFRvIII) in high grade astrocytomas [27], providing a possible target for immunotherapy [41]. Even though there are several studies advocating the importance of EGFR in the oncogenesis of astrocytomas [5,9], the prognostic role of EGFR overexpression and EGFR gene amplification is still not fully clarified [5,8,9,27,31,39,42-46]. Neither EGFR gene amplification nor EGFR overexpression was associated with survival in this study. Conflicting prognostic data may be related to various techniques and small series of tumors studied, so larger studies are required to explore the prognostic impact of EGFR.

C-*erb*B2 is already an established negative prognostic marker with an associated targeted therapy in human breast cancer [47]. It has been suggested that c-*erb*B2 is a preferred partner for heterodimerization with the other members of the EGFR family. The physiological ligand of c-*erb*B2 has not been identified, and it is possible that this receptor can undergo homo- and heterodimerization in the absence of ligand binding [48]. In our series of anaplastic astrocytomas, three different antibodies were applied for detection of the c-*erb*B2 receptor protein in tumor tissue. They were all reactive against the internal domain and provided satisfactory immunostainings. In line with divergent reports on the presence of this receptor in malignant astrocytomas [8,21,49-55], the number of immunoreactive tumors differed considerably between the antibodies used. This can partly be explained by the application of different antibodies, subjective assessments of immunostaining, differences in tissue processing, and small sample series. Accordingly, the reported prognostic power of this receptor protein is debatable as well. Even so, reports including the present study point to an unfavourable outcome in neuroepithelial tumors with c-*erb*B-2 overexpression [51,52,56,57].

Unlike breast cancer, gene amplification is not a prerequisite for overexpression in gliomas [49,58] and FISH analyses are therefore superfluous in this context.

The overexpression of c-*erb*B3 and c-*erb*B4 is in accordance with our previous study on glioblastomas as well as with others [8,21,54,59,60]. These findings suggest involvement of these two receptor proteins in the oncogenesis of astrocytomas. However, no association with survival was detected in our study. By forming heterodimers with other members of the EGFR family, c-*erb*B3 channels *erb*B signaling through the PI3K/Akt pathway which in turn promotes tumor growth and progression. This phenomenon may limit the impact of exclusively targeting EGFR and c-*erb*B2 proteins, and may explain the acquired resistance to anticancer drugs that inhibit receptor tyrosine kinases [61]. The close integration of different *erb*B signaling systems and their interactions with other tyrosine kinases such as IGF1-R and c-met, suggest that the members of the EGFR family should be dealt with as a group rather than individually.

The role of c-*erb*B4 in tumorigenesis has not been fully established. Cooperation between *erb *proteins may also be of clinical relevance as mutual expression of c-*erb*B2 and c-*erb*B4 serves as an independent negative prognostic factor in childhood medulloblastoma [56]. A unique feature for c-*erb*B4 is that pre-mRNA undergoes alternative splicing, generating structurally distinct isoforms. The c-*erb*B4 isoforms have different signalling capabilities and may lead to diverse cellular responses. This might explain the inconsistent reports evaluating the role of c-*erb*B4 in breast cancer [62].

This retrospective study has limitations typical of immunohistochemical approaches, including limited technical reproducibility and subjective interpretation. In summary, this study shows the convenience and feasibility of immunohistochemistry when determining the expression of receptor proteins in tumor tissue. Our findings indicate that c-*erb*B2 overexpression predicts aggressive behaviour in human anaplastic astrocytomas. The coexpression of members of the EGFR family underlines their role in the oncogenesis of anaplastic astrocytomas and supports their promising clinical relevance in regard to prognosis, diagnosis, and therapy.

## Abbreviations

EGFR: Epidermal growth factor receptor; FISH: Fluorescence in situ hybridization; KPS: Karnofsky performance status; GTR: Gross total resection; MRI: Magnetic resonance imaging; WHO: World Health Organization; N.D.: No data.

## Competing interests

SG has received grants from *Sparebanken Møre *and *The Family Blix Fund for medical research*. The other authors have no competing interests.

## Authors' contributions

SG carried out the experiments, collected and interpretated the data, and wrote the manuscript. BY carried out the experiments and wrote the manuscript. USG carried out the experiments and wrote the manuscript. MG collected data and wrote the manuscript. SL analyzed the data and wrote the manuscript. SHT contributed conception, designed the study, and wrote the manuscript. All authors read and approved the final manuscript.
